# 

**Published:** 1892-03

**Authors:** 


					﻿Vol. XII.
MARCH, 1892.
No.
THE
HOMEOPATHIC pHY^lClA]\l,
A Monthly Journal of Homoeopathic Materia Medica
and Clinical Medicine.
‘'He is a freeman whom truth makes free, and all are slaves beside."
u Seek the TYuth : come whence it may, cost what it wilt."
EDITKD AND PUBLISH KD BY
WALTER NI. JAMES, M. I).
PHILADELPHIA :
No. 1185 8PRUCK STREET.
L O NDO IT :
ALFRED HEATH & CO., Sole Agents for Great Britain,
114 Ebury Street, Eaton Square.
SSrYearh/ Subscription, $2.50. invariably in advance, Single numbers,' 26 cts.
lotered at the Philadelphia Pact-Office ai 8eoonJ*Claag Mail Matter.
				

